# Are Uterine and Ovarian Artery Doppler Velocimetry Values Good Pregnancy Predictors in Clomiphene Citrate Cycles?

**DOI:** 10.22074/ijfs.2015.4207

**Published:** 2015-04-21

**Authors:** Ali Irfan Guzel, Selcuk Erkılınc, Irfan Ozer, Aytekin Tokmak, Ayse Kurt Sahin, Mustafa Ugur

**Affiliations:** Division of Infertility and Gynecological Endocrinology, Department of Obstetrics and Gynecology, Dr. Zekai Tahir Burak Women’s Health Research and Education Hospital, Ankara, Turkey

**Keywords:** Clomiphene Citrate, Infertility, Uterine Artery, Ovarian, Doppler Velocimetry

## Abstract

**Background:**

We conducted this prospective study to evaluate the prognostic significance of uterine and ovarian artery Doppler velocimetry in clomiphene citrate (CC) cycles.

**Materials and Methods:**

A total of 80 patients with unexplained infertility were given 100 mg/day of CC from day 3 to day 7 of their cycles in this current prospective study. On cycle day 3, before administration of CC, each patient underwent Doppler transvaginal ultrasonography. The Doppler velocimetries of the right and left uterine and ovarian arteries were recorded and analyzed in association with demographic and clinical parameters.

**Results:**

TheThere were 6 out of 80 patients who became pregnant, the overall pregnancy rate in this population was 7.5% for the current study. The cases were divided into two groups according to whether they became pregnant or not. Demographic characteristics showed no statistically significant differences between these groups (p>0.05). However, the duration of infertility did show statistically significant differences between the groups. Doppler velocimetry was not statistically significantly different between the two groups.

**Conclusion:**

Doppler velocimetry of the uterine and ovarian arteries is not a factor in the prognosis for pregnancy in CC cycles.

## Introduction

Clomiphene citrate (CC) is an agent that has been used since the 1960s for ovulation induction. In appropriately selected anovulatory women, CC successfully induces ovulation in about 70-80% of cases; among these women the overall cycle fecundability is approximately 15% (1). CC is generally used as the first step in treating early stage endometriosis, anovulation that is related to unexplained infertility and borderline male factor infertility with decreased requirements for close monitoring (2). CC acts on the central nervous system as an anti-estrogen and increases the pulse frequency of follicle stimulating hormone (FSH) and luteinizing hormone (LH), in order to induce ovulation. This anti-estrogen effect also has negative impacts on the endometrium and cervical mucus (3, 4).

Factors that affect pregnancy rates in CC cycles have been reported in previous studies. The ages of the women, longer duration times of their infertility and decreased ovarian reserves have been described as factors that indicate poor prognosis for pregnancy in CC cycles (5). The diameter of the leading follicle on the day before human chorionic gonadotropin (hCG) administration and the endometrial thickness (ET) have also been evaluated in relation to the success of CC cycles (6).

In addition, Doppler velocimetry studies have been conducted to evaluate the changes in blood flow in the uterus and ovaries during the normal menstrual cycle (7, 8). Ng et al. (9) reported their findings on the effects of Doppler velocimetry on the blood supply to the entire endometrium and the sub-endometrial region. These authors evaluated the role of endometrial and sub-endometrial vascularity in women who underwent frozen-thawed embryo transfer (FET) cycles and found that neither of these blood flow measures was a good predictor of pregnancy in FET cycles. Nakai et al. (10) also discussed their findings on uterine artery Doppler velocimetry in cycles stimulated by CC. Their study found lower endometrial perfusion.

The current study evaluated the effectiveness of Doppler velocimetries of the uterine and ovarian arteries as a predictor of successful pregnancy in CC cycles.

## Materials and Methods

### Study design and participants

This interventional, cross-sectional study was undertaken from May 2013 to June 2013 in the Department of Obstetrics and Gynecology, Division of Infertility and Gynecological Endocrinology at Dr. Zekai Tahir Burak Women’s Health Education and Research Hospital. This is a tertiary referral research hospital located in the central region of Turkey. It is a government-supported hospital where most of the health services are offered free of charge.

### Ethical considerations

The study was designed according to the Helsinki Declaration (11) and all patients gave written informed consent. The study was also approved by the Ethics Research Committee of Dr Zekai Tahir Burak Women’s Health Education and Research Hospital (Ankara, Turkey).

### Data collection

Women who had had unexplained infertility (primary or secondary) for at least one year were included in the study. A total of 80 women received oral CC, 100 mg daily, from day 3 to day 7 of their menstrual cycles. All of the cases received regular ultrasound monitoring for follicle growth and ET through transvaginal ultrasonography on day 12 of their cycles and again every other day until the leading follicle reached 17-23 mm, triggered by hCG. Transvaginal Doppler velocimetry waveforms were also obtained from the bilateral uterine and ovarian arteries of each patient for analysis. The ultrasound examinations, performed on day 3 of the menstrual cycle while the patients were in the lithotomy position used color Doppler sonography with a 7.5 MHz pulse endovaginal Doppler system for blood flow analysis (Aloka Co., Tokyo, Japan). The women’s bladders were emptied before their examinations. The uterine artery was located laterally to the isthmic region of the uterus and the ovarian artery was found at the lateral pole of the ovary near the infundibulopelvic ligament.

The following factors that affected patients’ risk levels were recorded: age, infertility type, duration of infertility, gravidy, parity, number of pregnancy losses, number of living children, ET, cycle day 3 serum FSH, LH and estradiol (E2) levels, uterine and ovarian artery Doppler velocimetries, number of antral follicules, dominant follicle sizes and pregnancy. The Doppler velocimetries measured were the right side uterine and ovarian artery pulsatility index (PI) and resistance index (RI) in addition to the left side uterine and ovarian artery PI and RI.

The cases were divided into two groups according to whether patients achieved pregnancy or not. At the end of the study, a total of 6 (7.5%) patients were pregnant. Pregnancy was determined by positive β-hCG and a visible intrauterine gestational sac.

### Statistical analyses

The primary outcome measure was a clinical pregnancy. Continuous variables were not normally distributed and recorded as median (interquartile range) unless otherwise indicated. The normal distribution of the variables was analyzed by the Kolmogorov-Smirnov test. Statistical comparison was carried out by Chi-square (χ^2^), Mann-Whitney and independent sample t tests where appropriate. The sample size was determined according to the results of the central limit theorem (12). Statistical analysis was performed using the Statistical Program for Social Sciences (SPSS, Version 15.0, Chicago, IL, USA). The two-tailed value of p<0.05 was considered statistically significant.

## Results

The demographic and clinical characteristics of the cases are shown in table 1. The study included 80 patients who had had unexplained infertility for at least one year. The pregnancy rate was 7.5%. The mean age of the cases was 26.83±4.95 years for the group that achieved pregnancy and 27.45 ±5.53 years for the group that did not (p=0.789). There were 54 (67.5%) cases of primary infertility. The median (min-max) gravidy and parity of the cases were 0.41 (0-3) and 0.23 (0-2), respectively. The infertility type (primary or secondary) showed no statistically significant differences between the groups in terms of pregnancy rate (p=0.640). The mean duration of infertility was 2.58±0.80 years for the group that achieved pregnancy and 3.93±2.34 years for the group that did not. These values showed statistically significant differences between the groups (p=0.007). The ET on day 3 of the cycle was 1.83±0.98 mm in the group that achieved pregnancy and 2.37±1.02 mm in group that did not, with no statistically significant differences (p=0.241).

**Table 1:** The differences in demographic and clinical characteristics among the pregnant and non-pregnant groups


	Pregnant group	Non-pregnant group	P (95% CI)
	n=6	n=74	

**Age (Y) X±SD**	26.83±4.95	27.45±5.53	0.789(-5.27- 4.02)
*Gravidy ≤1	69	6	0.912	
*Parity ≤1	73	6	0.732	
Infertility type*			0.964	
Primary	4 (66, 66)	50 (67.6)		
Secondary	2 (33, 33)	24 (32.4)		
**Duration of infertility (Y)**	2.58±0.80	3.93±2.34	0.007(-3.28-0.57)	
**ET (mm)**	1.83±0.98	2.37±1.02	0.241(-1.41-0.32)	
**Antral follicle number**	6.66±3.72	6.76±3.15	0.947	
**Leading follicle (mm)**			0.501	
Right	16.50±6.44	15.16±7.30		
Left	15.16±4.51	14.77±4.82		
**FSH (mIU/ml)**	6.47±1.61	6.47±2.19	0.992(-1.82-1.83)	
**LH (mIU/ml)**	7.19±5.32	5.75±3.73	0.380(-1.81-4.70)	
**E2 (pg/ml)**	55.81±18.72	46.34±22.49	0.320(-9.35-28.28)	


CI; Confidence interval, *; P values calculated by χ2 test, FSH; Follicle stimulating hormone, LH; Luteinizing hormone, E2; Estradiol and ET; Endometrial thickness.

The differences between the basal blood hormone levels of the FSH, LH and E2 values were also not statistically significant between the groups (p>0.05). However, the durations of infertility of the two groups showed statistically significant differences (p<0.007). The basal follicle numbers were 6.66±3.72 for the right ovary and 6.75±3.15 for the left ovary in the group that achieved pregnancy and 6.33±4.08 (right ovary) and 6.67±3.17 (left ovary) for the group that did not. The mean diameters of the leading follicle on the day that hCG was administered for the group that achieved pregnancy was 16.50±6.44 mm and for the group that did not achieve pregnancy it was 15.16±4.51 mm, for the right ovary. For the left ovary, this value was 15.16±7.30 mm in the group that achieved pregnancy versus 14.77±4.82 mm in the group that did not achieve pregnancy (p>0.05).

The measurement of Doppler velocimetry on day 3 of the cycle was as follows: the right side uterine artery mean (min-max) was PI: 3.33 ± 2.40, RI: 1.14±0.59, the right side ovarian artery was PI: 2.30±1.97, RI: 1.11±0.87; the left side uterine artery was PI: 3.11±1.91, RI: 1.21 ±0.64 and the left side ovarian artery was PI: 3.29±2.31, RI: 1.10±0.72.

Table 2 shows the Doppler velocimetry values of the two groups. There were no statistically significant differences between the groups (p>0.05).

**Table 2:** Doppler velocimetries of uterine and ovarian arteries among the pregnant and non-pregnant groups


	Pregnant group	Non-pregnant group	P (95% CI)
	n=6	n=74	

**Right uterine artery PI**	2.46±0.66	3.40±2.48	0.294 (-2.98-1.08)
**Right uterine artery RI**	0.84±1.16	0.85±0.60	0.155 (-0.82-0.16)
**Right ovarian artery PI**	2.91±1.83	2.30±1.99	0.985 (-1.63-1.71)
**Right ovarian artery RI**	1.21±0.56	1.11±0.90	0.420 (-0.63-0.85)
**Left uterine artery PI**	2.59±0.94	3.16±1.97	0.406 (-2.18-1.06)
**Left uterine artery RI**	1.04±0.20	1.23±0.66	0.883 (-0.73-0.35)
**Left ovarian artery PI**	3.29±1.87	3.29±2.35	0.763 (-2.02-2.01)
**Left ovarian artery RI**	0.97±1.12	0.98±0.70	0.985 (-0.77-0.48)


CI; Confidence interval, PI; Pulsatility index and RI; Resistance index.

## Discussion

In this study we examined 80 women who had unexplained infertility for at least one year with the intent to evaluate the uterine and ovarian artery Doppler velocimetries as predictors of a successfully achieved pregnancy. The patients received oral CC, 100 mg daily, from day 3 to day 7 of their menstrual cycles. At the end of the study, 6 (7.5%) of the patients had achieved pregnancy. We divided the cases into two groups based on whether the patient had achieved pregnancy or not and found that the demographic and clinical parameters showed no statistically significant differences between the groups. However, the differences between the groups in terms of the durations of infertility were statistically significant. The Doppler velocimetry values also showed no statistically significant differences between the groups.

Doppler velocimetry of the uterine, spiral and ovarian arteries has been previously investigated in different types of cases, various phases of the menstrual cycle, ovulation induction and in early pregnancy. First, Kupesic and Kurjak (13) reported that blood flow in the uterine, spiral and ovarian arteries has a predictive value in endometrial receptivity. Bassil et al. (14) have also demonstrated that the RI values of the ovarian artery are a good indicator of ovarian inhibition and may be used to assess the optimal timing for the beginning of human menopausal gonadotrophin. In addition, Dickey et al. (15) have found impedance of the spiral artery to be a predictive parameter in the early development of the embryo. Based on these findings, we decided to conduct a study to evaluate the Doppler velocimetry in CC cycles.

Nakai et al. (10) studied the Doppler velocimetry values for spontaneous and stimulated menstrual cycles in women with unexplained infertility and found that the uterine blood flow was higher in spontaneous cycles than in cycles stimulated with CC.

In addition, Ragni et al. (16) evaluated 318 cases that received mild controlled ovarian stimulation (COS) and intrauterine insemination (IUI). They assessed the vascularity of the dominant follicle by Doppler ultrasound. These authors reported that Doppler velocimetry of follicular vascularity did not predict the chances for pregnancy in COS and IUI cycles. The findings of the current study were similar to this study; we have also believed that Doppler velocimetry of the uterine and ovarian arteries are not good predictors for achieving pregnancy.

In a previous study, Ng et al. (17) reported that the Doppler velocimetry values of uterine and ovarian arteries showed no differences between the early follicular phase and an ensuing CC challenge test.

Baruah et al. (18) designed a study with COS patients using letrozole or CC and studied the Doppler velocimetry of the spiral arteries. The study found that the letrozole group showed significantly lower impedance than the CC group and determined that the Doppler velocimetry of spiral arteries was a supportive tool for evaluating endometrial response in the treatment of anovulatory polycystic ovary syndrome patients with infertility.

## Conclusion

We believe that Doppler velocimetries of the uterine and ovarian arteries are not prognostic factors for pregnancy in CC cycles.
